# Effect of Interleukin 6 Deficiency on Renal Interstitial Fibrosis

**DOI:** 10.1371/journal.pone.0052415

**Published:** 2012-12-18

**Authors:** Jun Yang, Jiyuan Chen, Jingyin Yan, Liping Zhang, Gang Chen, Liqun He, Yanlin Wang

**Affiliations:** 1 Division of Nephrology, Department of Medicine, Baylor College of Medicine, Houston, Texas, United States of America; 2 Department of Urology, Union Hospital, Tongji Medical College, Huazhong University of Science and Technology, Wuhan, Hubei, China; 3 Department of Nephrology, Shuguang Hospital, Shanghai, China; Biomedical Research Foundation of the Academy of Athens, Greece

## Abstract

Our recent studies have shown that bone marrow-derived fibroblast precursors contribute significantly to the pathogenesis of renal fibrosis. However, the molecular mechanisms underlying the recruitment and activation of bone marrow-derived fibroblast precursors are incompletely understood. We found that interleukin 6 was induced in the kidney in a murine model of renal fibrosis induced by unilateral ureteral obstruction. Therefore, we investigated if interleukin 6 play a role in the recruitment and maturation of bone marrow-derived fibroblast precursors in the kidney during the development of renal fibrosis. Wild-type and interleukin 6 knockout mice were subjected to unilateral obstructive injury for up to two weeks. Interleukin 6 knockout mice accumulated similar number of bone marrow-derived fibroblast precursors and myofibroblasts in the kidney in response to obstructive injury compared to wild-type mice. Furthermore, IL-6 knockout mice expressed comparable α-SMA in the obstructed kidney compared to wild-type mice. Moreover, targeted disruption of Interleukin 6 did not affect gene expression of profibrotic chemokine and cytokines in the obstructed kidney. Finally, there were no significant differences in renal interstitial fibrosis or expression of extracellular matrix proteins between wild-type and interleukin 6 knockout mice following obstructive injury. Our results indicate that interleukin 6 does not play a significant role in the recruitment of bone marrow-derived fibroblast precursors and the development of renal fibrosis.

## Introduction

Renal fibrosis is the final common manifestation of chronic kidney disease leading to ESRD [1], [2]. Furthermore, tubulointerstitial fibrosis is a key structural component of obstructive nephropathy, which is the major cause of chronic kidney disease in children [3]. Renal interstitial fibrosis is characterized by fibroblast activation and excessive production and deposition of extracellular matrix (ECM), which results in the destruction and collapse of renal parenchyma and causes progressive loss of kidney function. Because activated fibroblasts are the principal effector cells responsible for ECM production, their activation is regarded as a key event in the pathogenesis of renal fibrosis [4]–[6]. Recent evidence indicates that these activated fibroblasts may originate from bone marrow-derived fibroblast progenitor cells [7]–[11].

Bone marrow-derived fibroblast precursors termed fibrocytes are derived from a subpopulation of monocytes via monocyte-to-fibroblast transition [12]–[15]. These cells express mesenchymal markers such as collagen I and vimentin and hematopoietic markers such as CD45 and CD11b [12], [16]–[18]. These cells in culture display an adherent, spindle-shape morphology and express α-SMA that is enhanced when cells are treated with TGF-β1, consistent with the concept that they can differentiate into myofibroblasts [16]–[18]. The differentiation of these cells is regulated by cytokines. Profibrotic cytokines – IL-4 and IL-13 promote myeloid fibroblast differentiation, whereas antifibrotic cytokines – IFN-γ and IL-12 inhibit its differentiation [14], [19]. Our recent study provides evidence that accumulation of myeloid fibroblast precursors in the kidney and development of renal fibrosis required chemokine CXCL16 induction in the renal tubular epithelial cells in a murine model of renal fibrosis induced by unilateral ureteral obstruction [10]. However, the molecular mechanisms underlying the recruitment and activation of these cells into injured kidneys are not fully understood.

Interleukin 6 (IL-6) is a multifunctional cytokine that has both pro- and anti-inflammatory properties [20]. Studies have shown that IL-6 is elevated in patients with chronic kidney disease [21]. However, the role of IL-6 in the pathogenesis of renal fibrosis is unknown. In the present study, we investigated the role of IL-6 in a murine model of renal fibrosis induced by unilateral ureteral obstruction (UUO) using IL-6 knockout (KO) mice. Our results showed that IL-6 deficiency has no significant effect on the uptake of myeloid fibroblasts and the development of renal fibrosis.

## Materials and Methods

### Animals

Animal experiments were approved by the Institutional Animal Care and Use Committee of Baylor College of Medicine (IACUC permit #: AN-5011). The investigation conforms with the recommendations in the Guide for the Care and Use of Laboratory Animals published by the US National Institutes of Health (NIH Publication No. 85–23, revised 1996). All efforts were made to minimize suffering. The IL-6 KO mice on a background of C57BL/6J were purchased from the Jackson Laboratory. Male WT or IL-6 KO mice at 8–10 weeks old age, weighing 20–30 g were anesthetized by i.p. injection of ketamine (80 mg/kg) and xylazine (10 mg/kg). Through a flank incision, the left ureter was exposed and completely ligated using fine suture material (4–0 silk) at two points [10]. Mice were allowed to recover from anesthesia and were housed in standard rodent cages with *ad libitum* access to water and food until sacrificed.

### Renal Morphology

Mice were euthanized and perfused by injections of PBS into the left ventricle of the heart to remove blood. One portion of the kidney tissue was fixed in 10% buffered formalin and embedded in paraffin, cut at 4 µm thickness, and stained with picrosirius red to identify collagen fibers. The picrosirius red-stained sections were scanned using a microscope equipped with a digital camera (Nikon, Melville, NY), and quantitative evaluation was performed using NIS-Elements Br 3.0 software. The collagen-stained area was calculated as a percentage of the total area.

### Quantitative Real-Time RT-PCR

Quantitative analysis of the target mRNA expression was performed with real-time reverse transcription – polymerase chain reaction (RT-PCR) by the relative standard curve method [10]. Total RNA was extracted from snap-frozen kidney tissues with TRIzol Reagent (Invitrogen). Total RNA were reverse-transcribed and amplified in triplicate using IQ SYBR green supermix reagent (Bio-Rad, Herculus, CA) with a real-time PCR machine (Bio-Rad, Herculus, CA), according to the manufacturer's instructions. The specificity of real-time PCR was confirmed by melting-curve analysis. The expression levels of the target genes were normalized to the GAPDH level in each sample. The following are the primer sequences: IL-6: Forward 5′- GAGGATACCACTCCCAACAGACC-3′ and reverse 5′- AAGTGCATCATCGTTGTTCATAC-3′; TGF-β1: Forward 5′- CAACAATTCCTGGCGTTACCTTGG-3′ and reverse 5′- GAAAGCCCTGTATTCCGTCTCCTT-3′; CXCL16: Forward 5′- ACCCTTGTCTCTTGCGTTCTTCCT-3′ and reverse 5′- ATGTGATCCAAAGTACCCTGCGGT-3′; IL-4: Forward 5′- ATCGGCATTTTGAACGAGGTC-3′ and reverse 5′- GAGGACGTTTGGCACATCCA-3′; IL-13: Forward 5′-CAGCCTCCCCGATACCAAAAT-3′ and reverse 5′- GCGAAACAGTTGCTTTGTGTAG-3′; GAPDH: Forward 5′-TGCTGAGTATGTCGTGGAGTCTA-3′ and reverse 5′-AGTGGGAGTTGCTGTTGAAATC-3′.

### Immunohistochemistry

Immunohistochemical staining was performed on paraffin sections. Antigen retrieval was performed with antigen unmasking solution (Vector Laboratories, Burlingame, CA). Endogenous peroxidase activity was quenched with 3% H_2_O_2_. After blocking, slides were incubated with primary antibody in a humidified chamber overnight. After washing, slides were incubated with appropriate secondary antibody and ABC solution sequentially according to the Vectastain ELITE ABC kit (Vector Laboratories, Burlingame, CA). The reaction was visualized by incubation with DAB solution for an appropriate period of time. Slides were then counterstained with hematoxylin, dehydrated, and coverslipped. The images were acquired and analyzed by NIS Element software with Nikon microscope image system.

### Immunofluorescence

Renal tissues were embedded in OCT compound, snap-frozen on dry ice, cut at 5 µm thickness using a cryostat, and mounted on Superfrost Plus microscope slides. After fixation, nonspecific binding was blocked with serum-free protein block (DAKO). Slides were then incubated with goat anti-MCP-1 antibody (R&D Systems) followed by Alexa-488 conjugated donkey anti-goat antibody (Invitrogen), rabbit anti-collagen I antibody (Rockland) followed by Alexa-488 conjugated donkey anti-rabbit antibody (Invitrogen), rabbit anti-fibronectin antibody (Sigma) followed by Alexa-488 conjugated donkey anti-rabbit antibody (Invitrogen), or rabbit anti-α-SMA antibody (Abcam) followed by Alexa-488 conjugated donkey anti-rabbit antibody (Invitrogen). For double immunofluorescence, kidney sections were fixed and stained with primary antibodies followed by appropriate secondary antibodies sequentially. Slides were mounted with mounting medium with DAPI. Fluorescent intensity was visualized using a microscope equipped with a digital camera (Nikon, Melville, NY). Quantitative evaluation of sections stained with antibodies to α-SMA, collagen I and fibronectin was performed using NIS-Elements Br 3.0 software. The fluorescence-positive area was calculated as a percentage of the total area.

### Western Blot Analysis

Protein was extracted using the RIPA buffer containing cocktail proteinase inhibitors (Thermo Fisher Scientific Inc., Rockford, IL) and quantified with Bio-Rad protein assay. Equal amount of protein was separated on SDS–polyacrylamide gels in a Tris/glycine buffer system, transferred onto nitrocellulose membranes, and blotted according to standard procedures with primary antibodies overnight followed by incubation with appropriate fluorescence-conjugated secondary antibodies. The proteins of interest were analyzed using an Odyssey IR scanner, and signal intensities were quantified using NIH Image/J software.

### Statistical Analysis

All data were expressed as mean ± SEM. Multiple group comparisons were performed by one-way ANOVA followed by the Bonferroni procedure for comparison of means. *P*<0.05 was considered statistically significant.

## Results

### IL-6 Is Induced in a Mouse Model of Renal Fibrosis

**Figure 1:**
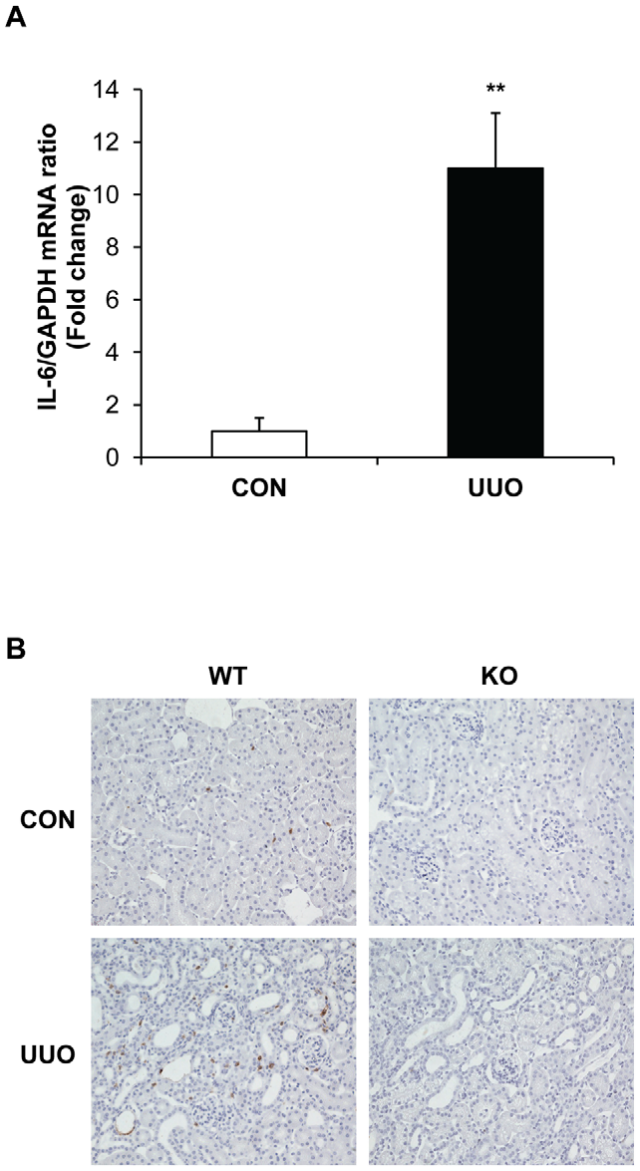
IL-6 is induced in the kidney following obstructive injury. **A.** Graphic presentation shows IL-6 mRNA induction. ** *P*<0.01 vs normal control kidney. n = 4. **B.** Representative photomicrographs of kidney sections stained for IL-6 (brown) and counter stained with hematoxylin (blue) (Original magnification: X400).

We examined if IL-6 is induced in the kidney in response to UUO. Using real time RT-PCR, we found that the mRNA level of IL-6 was markedly upregulated in obstructed kidneys compared with the contralateral control kidneys 7 days after surgery (Figure 1A). To identify the cell types responsible for IL-6 production in the kidney, serial sections of kidneys were stained with an IL-6 antibody. Our results revealed that IL-6 protein was localized mainly in the interstitial cells of obstructed kidneys (Figure 1B). Of note, no IL-6 positive staining was detected in IL-6 KO mice, indicating the specificity of the antibody against IL-6.

### IL-6 Deficiency Does not Affect Myeloid Fibroblast Accumulation

**Figure 2:**
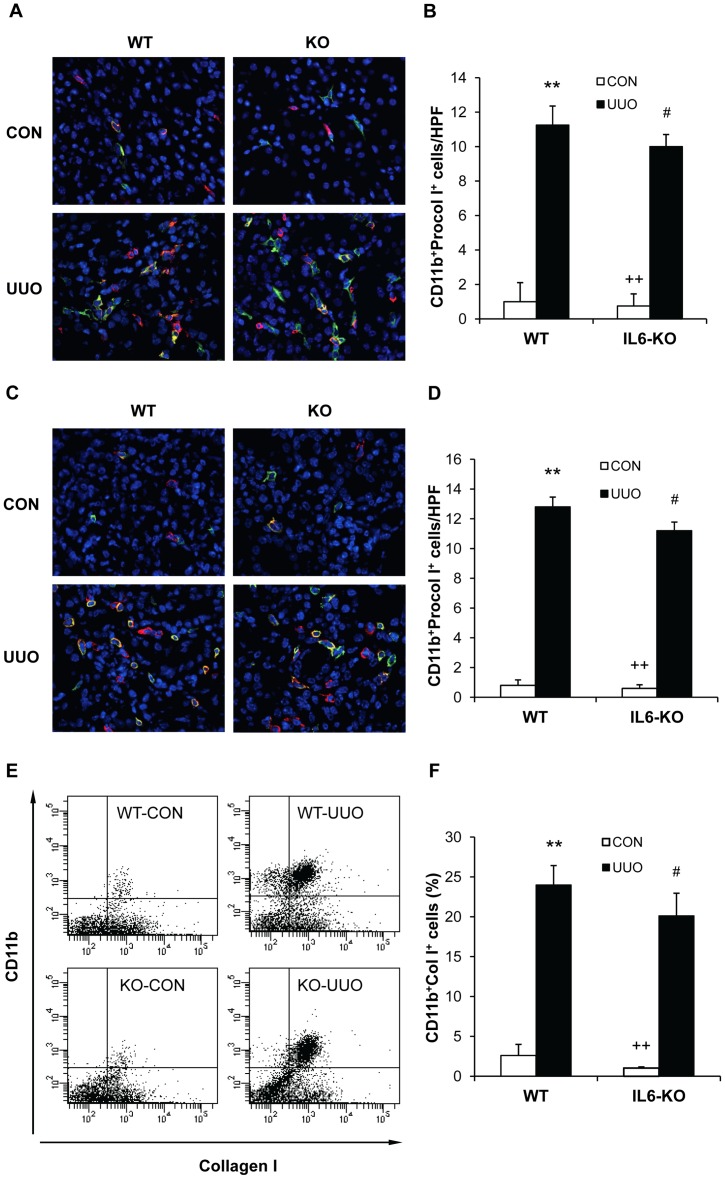
Effect of IL-6 deficiency on the accumulation of bone marrow-derived fibroblast precursors in the kidney after UUO. **A.** Representative photomicrographs of kidney sections from WT and IL-6 KO mice 5 days after UUO stained for CD11b (red), procollagen I (green), and DAPI (blue). **B.** Quantitative analysis of CD11b^+^ and procollagen I^+^ fibroblasts in the kidney of WT and IL-6 KO mice 5 days after UUO. ** *P*<0.01 vs WT control, ^#^ *P*>0.05 vs WT-UUO, and ^++^ *P*<0.01 vs KO-UUO. n = 4 per group. **C.** Representative photomicrographs of kidney sections from WT and IL-6 KO mice 1 week after UUO stained for CD11b (red), procollagen I (green), and DAPI (blue). **D.** Quantitative analysis of CD11b^+^ and procollagen I^+^ fibroblasts in the kidney of WT and IL-6 KO mice 1 week after UUO. ** *P*<0.01 vs WT control, ^#^ *P*>0.05 vs WT-UUO, and ^++^ *P*<0.01 vs KO-UUO. n = 5 per group. **E.** Representative cytometric diagrams showing the effect of IL-6 deficiency on the accumulation of CD11b and collagen I dual positive fibroblasts in the kidney of WT and IL-6 KO mice 1 week after UUO. **F.** Quantitative analysis of CD11b and collagen I dual positive fibroblasts in the kidney of WT and IL-6 KO mice 1 week after UUO. ** *P*<0.01 vs WT control, ^#^ *P*>0.05 vs WT UUO, and^ ++^ *P*<0.01 vs KO UUO. n = 3 per group.

To examine if IL-6 plays a role in the accumulation of bone marrow-derived fibroblasts in the kidneys, WT and IL-6 KO mice were subjected to obstructive injury for 5 or 7 days. Kidney sections were stained for CD11b and procollagen I and examined with a fluorescent microscope. Our results showed that both WT and IL-6 KO mice exhibited a marked increase in the number of CD11b^+^ and procollagen I^+^ fibroblasts in obstructed kidneys, which was not statistically different between these two groups (Figure 2, A–D). Consistent with immunofluorescent staining, flow cytometric analysis of freshly-isolated renal cells stained for CD11b and collagen I demonstrated that targeted disruption of IL-6 had no effect on the accumulation of CD45^+^ and collagen I^+^ fibroblasts in the kidneys compared with WT mice following UUO (Figure 2, E–F). These data indicate that IL-6 does not play an important role in the recruitment of bone marrow-derived fibroblasts into the kidney in response to obstructive injury.

### IL-6 Deficiency Does not Influence Myofibroblast Activation

**Figure 3:**
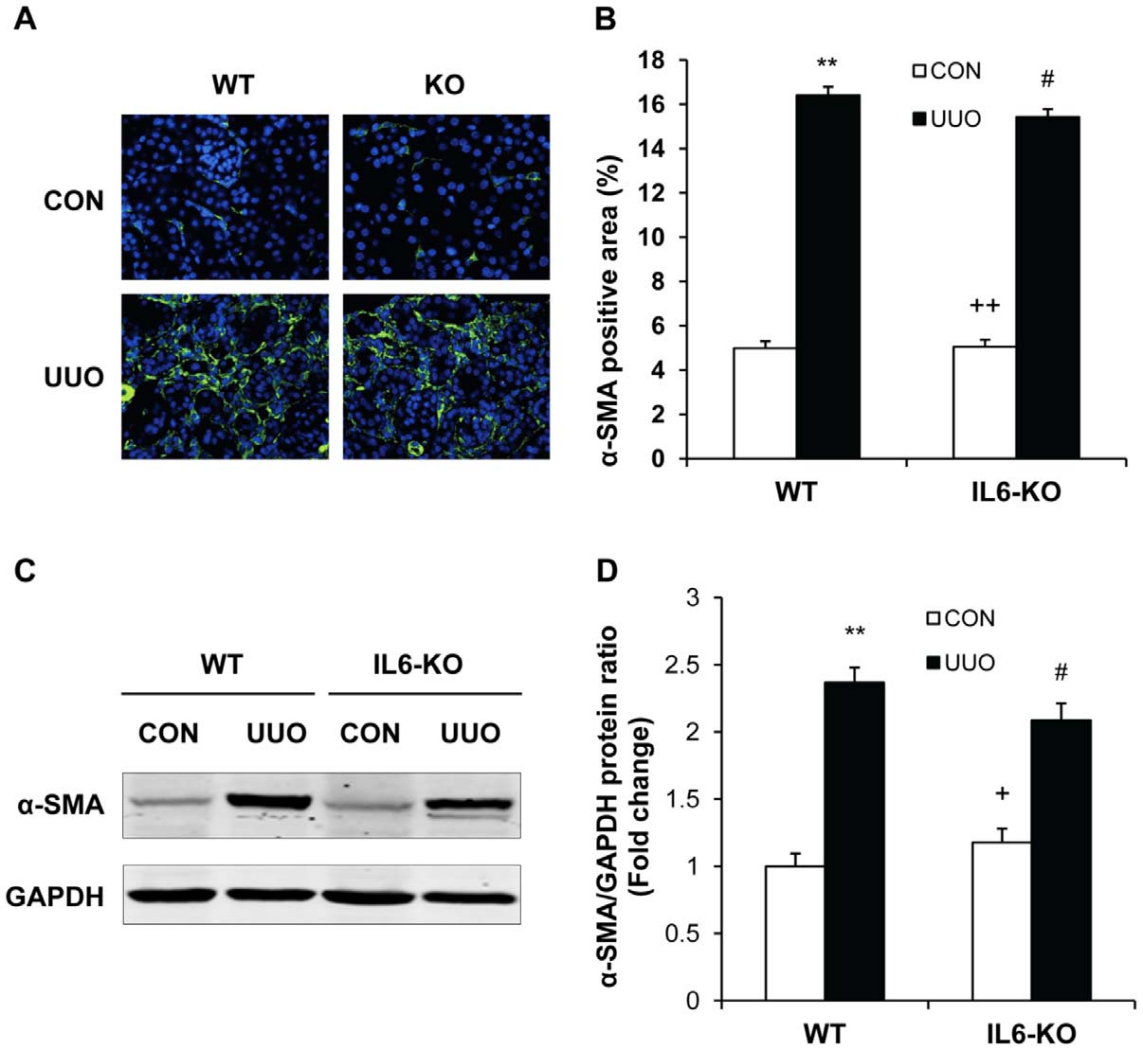
Effect of IL-6 deficiency on myofibroblast activation and α-SMA expression in obstructive nephropathy. **A.** Representative photomicrographs of α-SMA immunostaining in the kidney of WT and IL-6 KO mice after UUO. **B.** Quantitative analysis of α-SMA protein expression in the kidney of WT and IL-6 KO mice after UUO. ** *P*<0.01 vs WT controls; ^#^ *P*>0.05 vs WT UUO; ^++^ *P*<0.01 vs KO UUO. n = 5 per group. **C.** Representative Western blots show the levels of α-SMA protein expression in the kidney of WT and IL-6 KO mice. **D.** Quantitative analysis of α-SMA protein expression in the kidney of WT and IL-6 KO mice. ** *P<*0.01 vs WT controls; ^#^ *P>*0.05 vs WT UUO; ^+^ *P<*0.05 vs KO UUO. n = 3 per group.

To determine if IL-6 deficiency influences myofibroblast activation in the kidney, WT and IL-6 KO mice were subjected to UUO for 7 days. Kidney sections were stained with an antibody against α-SMA, a marker of myofibroblasts, and examined with a fluorescent microscope. The results revealed that targeted deletion of IL-6 did not alter myofibroblast activation in obstructed kidneys compared with WT mice (Figure 3, A–B). Consistent with these findings, Western blot analysis showed that both WT mice and IL-6 deficiency mice had similar increases in protein expression levels of α-SMA in the kidneys following obstructive injury (Figure 3, C–D). These results indicate that IL-6 does not regulate myofibroblast activation.

### IL-6 Deficiency Does not Affect Profibrotic Molecule Expression

**Figure 4:**
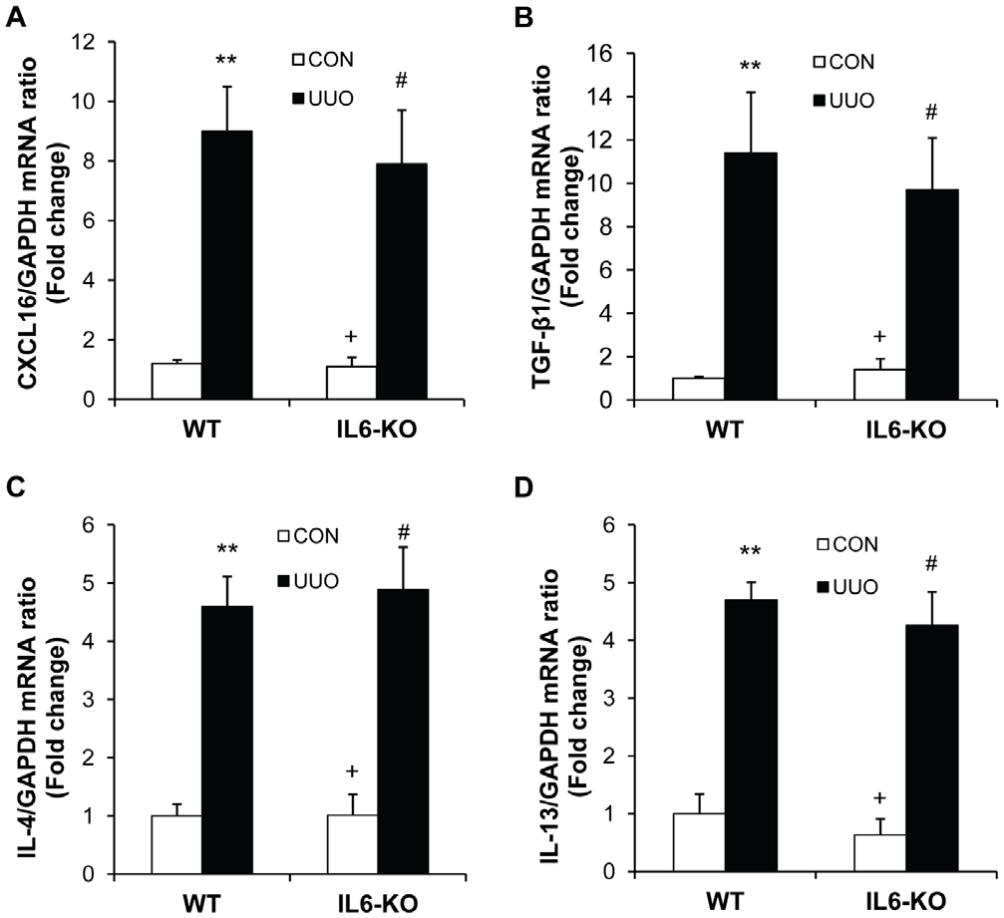
Effect of IL-6 deficiency on profibrotic molecule expression. **A.** IL-6 deficiency does not affect CXCL16 gene expression. The mRNA levels of CXCL16 in the kidneys of WT and IL-6 KO mice were determined by real-time RT-PCR. ** *P*<0.01 vs WT control, ^#^ *P*>0.05 vs WT UUO, and ^+^ *P*<0.05 vs KO UUO. n = 3–4 per group. **B.** IL-6 deficiency does not influence TGF-β1 gene expression. The mRNA levels of TGF-β1 in the kidneys of WT and IL-6 KO mice were determined by real-time RT-PCR. ** *P*<0.01 vs WT control, ^#^ *P*>0.05 vs WT UUO, and ^+^ *P*<0.05 vs KO UUO. n = 3–4 per group. **C.** IL-6 deficiency does not influence IL-4 gene expression. The mRNA levels of IL-4 in the kidneys of WT and IL-6 KO mice were determined by real-time RT-PCR. ** *P*<0.01 vs WT control, ^#^ *P*>0.05 vs WT UUO, and ^+^ *P*<0.05 vs KO UUO. n = 3–4 per group. **D.** IL-6 deficiency does not influence IL-13 gene expression. The mRNA levels of IL-13 in the kidneys of WT and IL-6 KO mice were determined by real-time RT-PCR. ** *P*<0.01 vs WT control, ^#^ *P*>0.05 vs WT UUO, and ^+^ *P*<0.05 vs KO UUO. n = 3–4 per group.

We have recently demonstrated that the presence and development of bone marrow-derived fibroblasts from mononuclear cells appear to be driven by and dependent upon induction of the chemokine, CXCL16, in renal tubular epithelial cells and is inhibited by genetic deletion of CXCL16 [10]. We therefore examined if IL-6 deficiency affects CXCL16 gene expression. The results of real time RT-PCR showed that targeted disruption of IL-6 did not significantly affect CXCL16 mRNA expression in the kidney in response to obstructive injury (Figure 4A). These data indicate that IL-6 signaling does not regulate chemokine CXCL16 gene expression in the kidney following obstructive injury.

TGF-β1 is a key cytokine that mediates myofibroblast activation during the development of renal fibrosis [22]–[25]. We determined if IL-6 deficiency influences TGF-β1 gene expression. The results of real time RT-PCR revealed that IL-6 deficiency did not affect TGF-β1 mRNA in the kidney following obstructive injury (Figure 4B). These data suggest that IL-6 signaling does not play a major role in the regulation of TGF-β1 gene expression in the kidney in response to obstructive injury.

Since Th2 cytokines – IL-4 and IL-13 have been shown to promote monocyte-to-fibroblast transition [14], [19], we then examined if IL-6 deficiency affects these cytokine expression in the kidney. The results of real time RT-PCR showed that IL-6 deficiency did not influence mRNA levels of IL-4 and IL-13 in the kidney following obstructive injury (Figure 4 C–D). These data indicate that IL-6 signaling does not modulate the gene expression of IL-4 and IL-13 in the kidney in response to obstructive injury.

### IL-6 Deficiency Has no Effect on Renal Fibrosis

**Figure 5:**
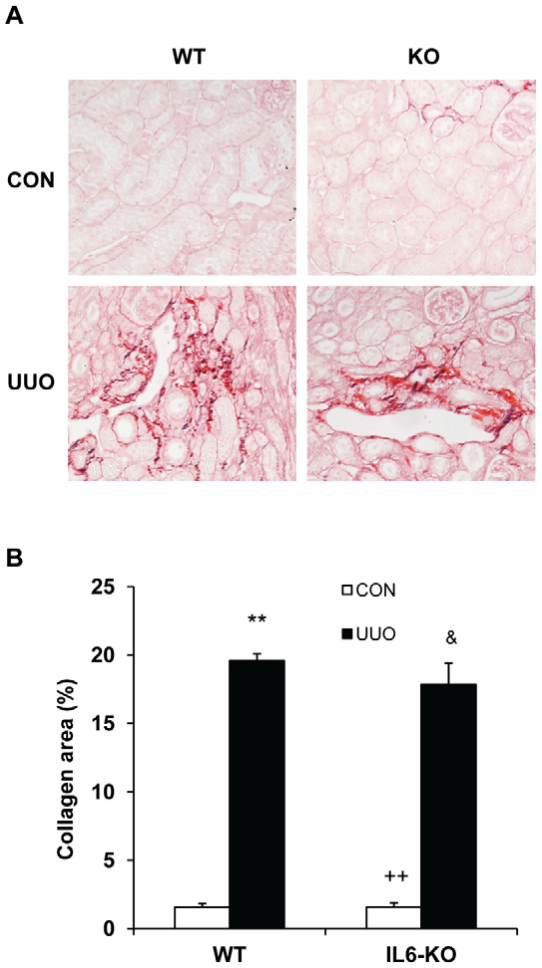
Effect of IL-6 deficiency on renal fibrosis and extracellular matrix deposition in the kidney. **A.** Representative photomicrographs show kidney sections stained with picrosirius red for assessment of total collagen deposition. **B.** Bar graph shows quantitative analysis of renal interstitial collagen in different groups as indicated. ** *P*<0.01 vs WT control, ^&^ *P*>0.05 vs WT UUO, and ^++^ *P*<0.01 vs KO UUO. n = 5 per group.

Since IL-6 does not regulate the accumulation and activation of bone marrow-derived fibroblasts in the kidney in response to obstructive injury, we then examined if IL-6 deficiency has an effect on the development of renal fibrosis. WT and IL-6 KO mice were subjected to UUO for 14 days. Both WT and IL-6 KO mice developed similar degree of collagen deposition in obstructed kidneys as demonstrated by picrosirius red staining (Figure 5). These data indicate that IL-6 does not play a role in the pathogenesis of renal fibrosis.

### IL-6 Deficiency Does not Affect ECM Protein Expression

**Figure 6:**
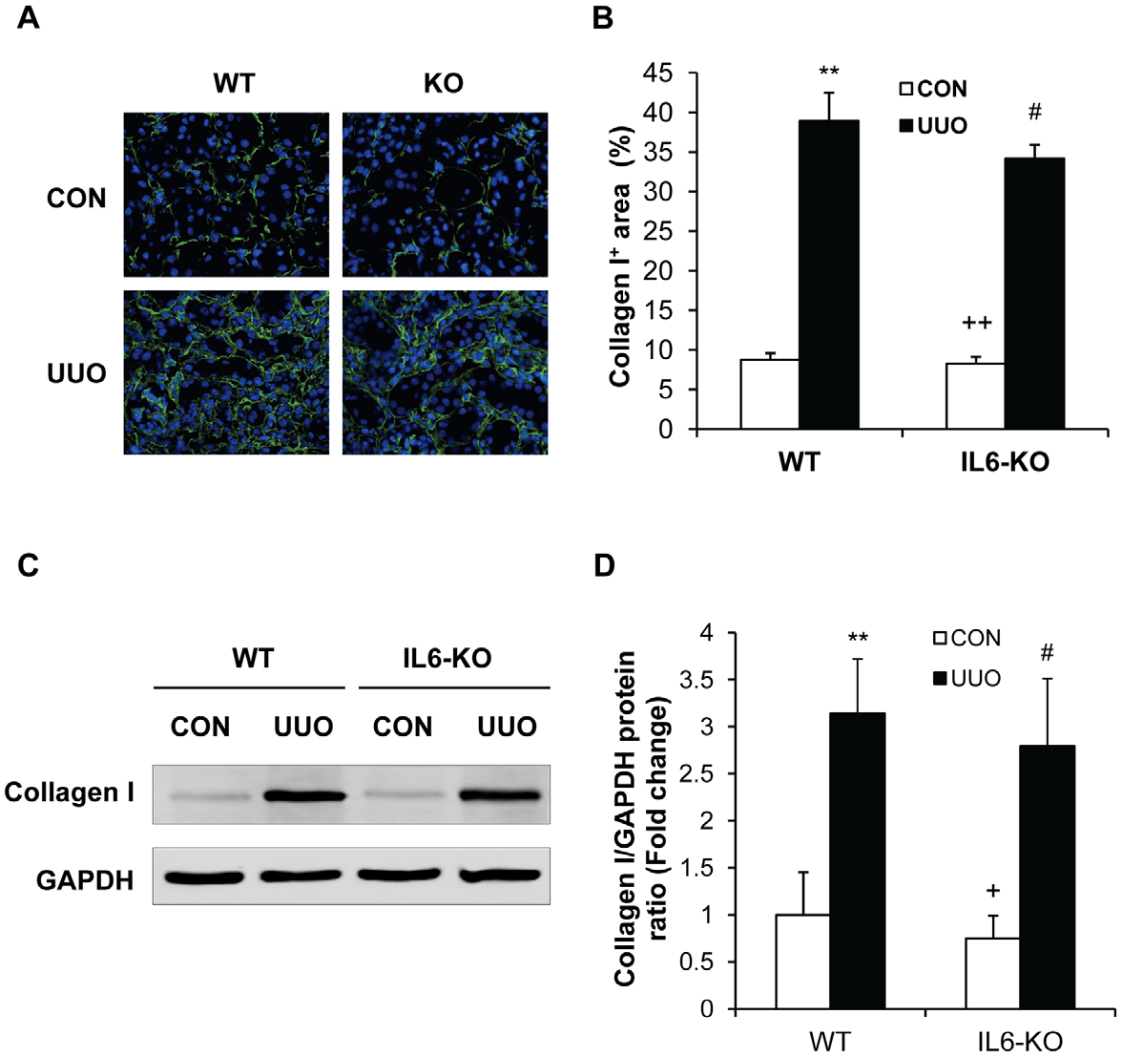
Effect of IL-6 deficiency on collagen I expression in the kidney. **A.** Representative photomicrographs of collagen I immunostaining in the kidney of WT and IL-6 KO mice after surgery (original magnification X400). **B.** Quantitative analysis of interstitial collagen I protein expression in the kidney sections of WT and IL-6 KO mice. ** *P*<0.01 vs WT-control, ^#^ *P*>0.05 vs WT UUO, and ^++^ *P*<0.01 vs KO UUO. n = 5 per group. **C.** Representative Western blots show the protein levels of collagen I in the kidney of WT and IL-6 KO mice. **D.** Quantitative analysis of collagen I protein expression in the kidney of WT and IL-6 KO mice. ** *P*<0.01 vs WT controls, ^#^ *P*>0.05 vs WT UUO, and ^+^ *P*<0.05 vs KO UUO. n = 4 per group.

**Figure 7:**
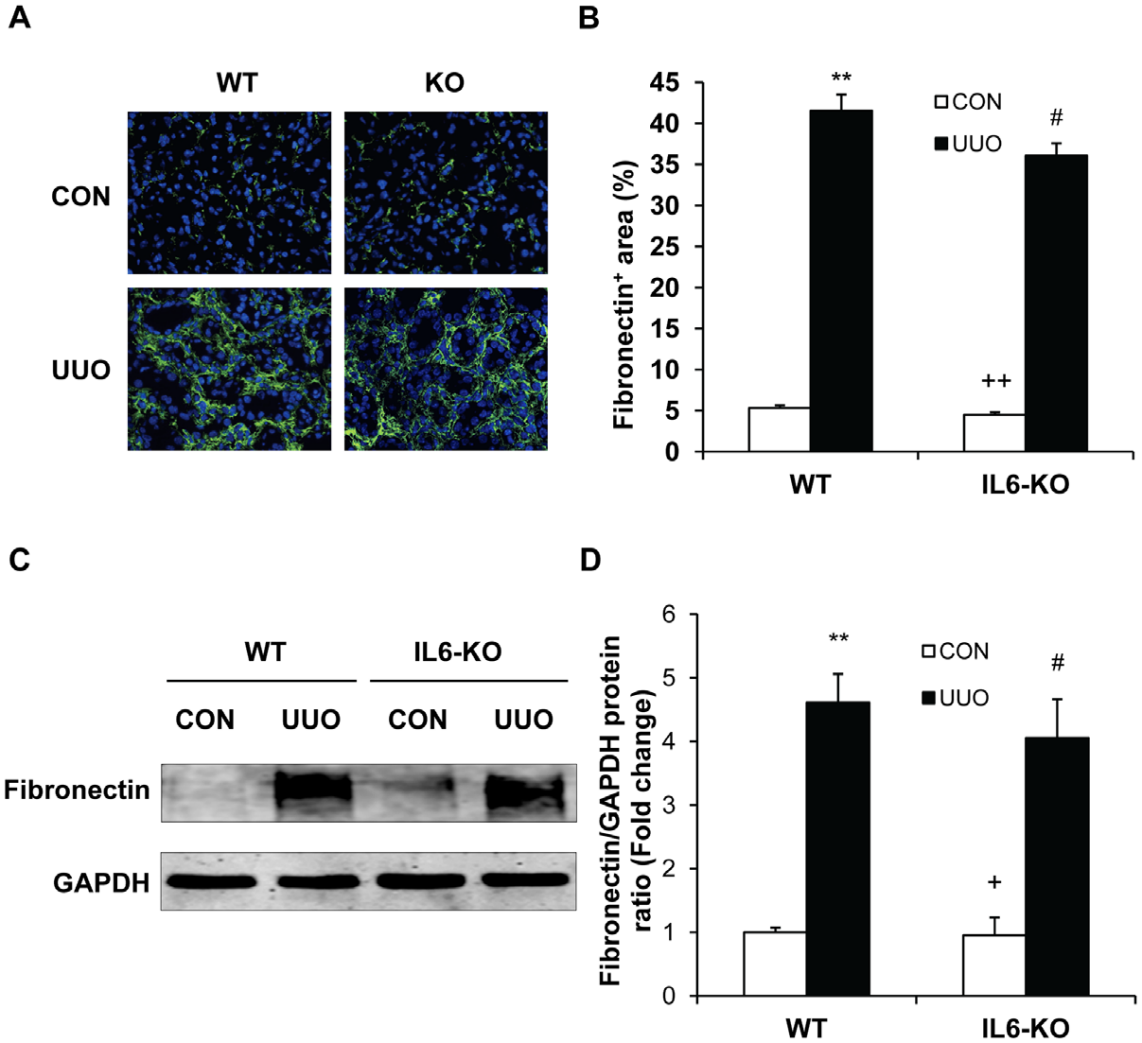
Effect of IL-6 deficiency on fibronectin expression in the kidney. **A.** Representative photomicrographs of fibronectin immunostaining in the kidney of WT and IL-6 KO mice after UUO (original magnification X400). **B.** Quantitative analysis of interstitial fibronectin protein expression in the kidney sections of WT and IL-6 KO mice. ** *P*<0.01 vs WT control, ^#^ *P*>0.05 vs WT UUO, and ^++^ *P*<0.01 vs KO UUO. n = 5 per group. **C.** Representative Western blots show the protein levels of fibronectin in the kidney of WT and IL-6 KO mice. **D.** Quantitative analysis of fibronectin protein expression in the kidney of WT and IL-6 KO mice. ** *P*<0.01 vs WT controls, ^#^ *P*>0.05 vs WT UUO, and ^+^ *P*<0.05 vs KO UUO. n = 4 per group.

We next investigated the effect of targeted disruption of IL-6 on the expression and accumulation of collagen I and fibronectin, two major components of ECM. Both WT and IL-6 KO mice displayed a marked increase in the protein expression levels of collagen I and fibronectin in the kidneys following obstructive injury, which was not statistically different between these two groups (Figure 6 and 7). These data indicate that IL-6 does not regulate the production and deposition of ECM proteins in the kidney following obstructive injury.

## Discussion

In this study, we demonstrate that (1) IL-6 is induced in the kidney in response to obstructive injury; (2) WT and IL-6 KO mice accumulate similar number of bone marrow-derived fibroblast precursors in the kidney following obstructive injury; (3) Targeted disruption of IL-6 has no significant effect on myofibroblast formation and α-SMA expression; (4) Targeted disruption of IL-6 does not influence gene expression of profibrotic chemokines and cytokines; (5) Targeted disruption of IL-6 does not alter the severity of renal fibrosis and the expression of ECM proteins. These results indicate that IL-6 does not play an important role in the recruitment of bone marrow-derived fibroblasts and the development of renal fibrosis induced by obstructive injury.

Renal fibrosis is a pathological hallmark of chronic kidney disease regardless of underlying etiologies. Activated fibroblasts are responsible for the excessive production of extracellular matrix. Recent studies have provided evidence that bone marrow-derived fibroblast precursors are recruited into the kidney and contribute significantly to the pathogenesis of renal fibrosis [7]–[10]. These cells express the hematopoietic markers such as CD11b and the mesenchymal markers such as collagen I. The signaling mechanisms underlying the recruitment of these bone marrow-derived fibroblast precursors into the kidney are incompletely understood.

IL-6 is a multifunctional cytokine that regulates inflammatory process. Studies have shown that targeted disruption of IL-6 attenuates acute kidney injury induced by ischemia-reperfusion [26] or mercury [27]. However, its role in the pathogenesis of renal interstitial fibrosis is unknown. In the present study, we demonstrate that targeted disruption of IL-6 does not affect the accumulation of bone marrow-derived fibroblasts expressing hematopoietic marker (CD11b) and mesenchymal marker (collagen I) in the kidney and the degree of renal fibrosis in a murine model of obstructive nephropathy. These data indicate that IL-6 does not play an important role for the recruitment of bone marrow-derived fibroblast precursors into the kidney in response to obstructive injury.

Myofibroblasts are a population of smooth muscle-like fibroblasts that play an important role in wound healing and organ fibrosis [28]. Myofibroblasts are regarded as the key cell types that are responsible for excessive production and deposition of extracellular matrix during the development of kidney fibrosis [4], [29]. Furthermore, both experimental and clinical studies have shown that the number of interstitial myofibroblasts correlates well with the severity of tubulointerstitial fibrosis and progression of kidney disease [30]–[32]. Our present study demonstrates that myofibroblasts identified as α-SMA positive cells accumulate in the kidney of WT mice following obstructive injury, and their accumulation does not alter significantly in the obstructed kidney of IL-6 KO mice. These results indicate that IL-6 does not influence myofibroblast formation in the kidney in response to obstructive injury.

Chemokine (C-X-C motif) ligand 16 (CXCL16) is a cytokine belonging to the CXC chemokine family [33]. There are two forms of CXCL16. The transmembrane form of CXCL16 is composed of a CXC chemokine domain, a mucin-like stalk, a transmembrane domain and a cytoplasmic tail. The soluble form of CXCL16 resulting from cleavage at the cell surface is composed of the extracellular stalk and the chemokine domain. The transmembrane form of CXCL16 functions as an adhesion molecule for CXCR6 expressing cells and scavenger receptor for oxidized low-density lipoprotein while the soluble form of CXCL16 functions as a chemoattractant to promote circulating cell migration into sites of injury [34], [35]. We have previously shown that CXCL16 is induced in the kidney in response to obstructive injury and plays a critical role in recruiting bone marrow-derived fibroblasts into kidney and the development of renal fibrosis in a murine model of chronic kidney disease induced by unilateral ureteral obstruction [10]. Our present results show that targeted disruption of IL-6 does not affect CXCL16 expression in the kidney. These results indicate that IL-6 deficiency does not play a role in the regulation of CXCL16 gene expression in the kidney in response to obstructive injury.

TGF-β1 is a profibrotic cytokine that plays an essential role in the activation of fibroblasts during the pathogenesis of renal fibrosis through activation of a cascade of intracellular signaling pathways [22]–[25]. Furthermore, IL-4 and IL-13 are profibrotic Th2 cytokines, which has been reported to play an important role in the pathogenesis of fibrosis through TGF-β1-dependent and independent mechanisms [36]–[38]. Our results reveal that targeted disruption of IL-6 does not affect the mRNA expression levels of TGF-β1, IL-4, and IL-13 in the kidney after obstructive injury compared with WT mice. These results are consistent with our observation that IL-6 deficiency does not significantly influence myeloid fibroblast activation in the kidney following obstructive injury.

A prominent feature of renal interstitial fibrosis is a striking increased production and deposition of extracellular matrix proteins such as collagens and fibronectin. Morphometric analysis of picrosirius red staining of kidney sections at day 14 after obstructive injury demonstrates the presence of interstitial collagen deposition. This collagen deposition is not significantly altered in the obstructed kidneys of IL-6 KO mice. Consistent with these findings, we further illustrate that both WT and IL-6 KO mice display similar increases in collagen I and fibronectin following obstructive injury. These data indicate that IL-6 signaling does not participate in the regulation extracellular matrix protein production and deposition.

In summary, our results demonstrate that IL-6 signaling does not play a significant role in the recruitment of bone marrow-derived fibroblasts into the kidney and the development of renal fibrosis induced by obstructive injury.

## References

[1] Schainuck LI, Striker GE, Cutler RE, Benditt EP (1970) Structural-functional correlations in renal disease. II. The correlations. Hum Pathol 1: 631–641. 5521736 10.1016/s0046-8177(70)80061-2

[2] Nath KA (1998) The tubulointerstitium in progressive renal disease. Kidney Int 54: 992–994. 9734628 10.1046/j.1523-1755.1998.00079.x

[3] Eddy AA (2000) Molecular basis of renal fibrosis. Pediatr Nephrol 15: 290–301. 11149129 10.1007/s004670000461

[4] Neilson EG (2006) Mechanisms of disease: Fibroblasts–a new look at an old problem. Nat Clin Pract Nephrol 2: 101–108. 16932401 10.1038/ncpneph0093

[5] Strutz F, Muller GA (2006) Renal fibrosis and the origin of the renal fibroblast. Nephrol Dial Transplant 21: 3368–3370. 16887850 10.1093/ndt/gfl199

[6] Liu Y (2006) Renal fibrosis: new insights into the pathogenesis and therapeutics. Kidney Int 69: 213–217. 16408108 10.1038/sj.ki.5000054

[7] Sakai N, Wada T, Yokoyama H, Lipp M, Ueha S, et al (2006) Secondary lymphoid tissue chemokine (SLC/CCL21)/CCR7 signaling regulates fibrocytes in renal fibrosis. Proc Natl Acad Sci U S A 103: 14098–14103. 16966615 10.1073/pnas.0511200103 PMC1599918

[8] Grimm PC, Nickerson P, Jeffery J, Savani RC, Gough J, et al (2001) Neointimal and tubulointerstitial infiltration by recipient mesenchymal cells in chronic renal-allograft rejection. N Engl J Med 345: 93–97. 11450677 10.1056/NEJM200107123450203

[9] Broekema M, Harmsen MC, van Luyn MJ, Koerts JA, Petersen AH, et al (2007) Bone marrow-derived myofibroblasts contribute to the renal interstitial myofibroblast population and produce procollagen I after ischemia/reperfusion in rats. J Am Soc Nephrol 18: 165–175. 17135399 10.1681/ASN.2005070730

[10] Chen G, Lin SC, Chen J, He L, Dong F, et al (2011) CXCL16 recruits bone marrow-derived fibroblast precursors in renal fibrosis. Journal of the American Society of Nephrology: JASN 22: 1876–1886. 21816936 10.1681/ASN.2010080881 PMC3187185

[11] Li J, Deane JA, Campanale NV, Bertram JF, Ricardo SD (2007) The contribution of bone marrow-derived cells to the development of renal interstitial fibrosis. Stem Cells 25: 697–706. 17170067 10.1634/stemcells.2006-0133

[12] Bucala R, Spiegel LA, Chesney J, Hogan M, Cerami A (1994) Circulating fibrocytes define a new leukocyte subpopulation that mediates tissue repair. Mol Med 1: 71–81. 8790603 PMC2229929

[13] Niedermeier M, Reich B, Rodriguez Gomez M, Denzel A, Schmidbauer K, et al (2009) CD4+ T cells control the differentiation of Gr1+ monocytes into fibrocytes. Proc Natl Acad Sci U S A 106: 17892–17897. 19815530 10.1073/pnas.0906070106 PMC2764893

[14] Shao DD, Suresh R, Vakil V, Gomer RH, Pilling D (2008) Pivotal Advance: Th-1 cytokines inhibit, and Th-2 cytokines promote fibrocyte differentiation. Journal of leukocyte biology 83: 1323–1333. 18332234 10.1189/jlb.1107782 PMC2659591

[15] Haudek SB, Trial J, Xia Y, Gupta D, Pilling D, et al (2008) Fc receptor engagement mediates differentiation of cardiac fibroblast precursor cells. Proc Natl Acad Sci U S A 105: 10179–10184. 18632582 10.1073/pnas.0804910105 PMC2465805

[16] Metz CN (2003) Fibrocytes: a unique cell population implicated in wound healing. Cell Mol Life Sci 60: 1342–1350. 12943223 10.1007/s00018-003-2328-0 PMC11138753

[17] Quan TE, Cowper S, Wu SP, Bockenstedt LK, Bucala R (2004) Circulating fibrocytes: collagen-secreting cells of the peripheral blood. Int J Biochem Cell Biol 36: 598–606. 15010326 10.1016/j.biocel.2003.10.005

[18] Abe R, Donnelly SC, Peng T, Bucala R, Metz CN (2001) Peripheral blood fibrocytes: differentiation pathway and migration to wound sites. J Immunol 166: 7556–7562. 11390511 10.4049/jimmunol.166.12.7556

[19] Cieslik KA, Taffet GE, Carlson S, Hermosillo J, Trial J, et al (2011) Immune-inflammatory dysregulation modulates the incidence of progressive fibrosis and diastolic stiffness in the aging heart. Journal of molecular and cellular cardiology 50: 248–256. 20974150 10.1016/j.yjmcc.2010.10.019 PMC3019252

[20] Gadient RA, Patterson PH (1999) Leukemia inhibitory factor, Interleukin 6, and other cytokines using the GP130 transducing receptor: roles in inflammation and injury. Stem Cells 17: 127–137. 10342555 10.1002/stem.170127

[21] Pecoits-Filho R, Lindholm B, Axelsson J, Stenvinkel P (2003) Update on interleukin-6 and its role in chronic renal failure. Nephrol Dial Transplant 18: 1042–1045. 12748331 10.1093/ndt/gfg111

[22] Border WA, Noble NA (1994) Transforming growth factor beta in tissue fibrosis. N Engl J Med 331: 1286–1292. 7935686 10.1056/NEJM199411103311907

[23] Border WA, Okuda S, Languino LR, Sporn MB, Ruoslahti E (1990) Suppression of experimental glomerulonephritis by antiserum against transforming growth factor beta 1. Nature 346: 371–374. 2374609 10.1038/346371a0

[24] Bottinger EP, Bitzer M (2002) TGF-beta signaling in renal disease. Journal of the American Society of Nephrology: JASN 13: 2600–2610. 12239251 10.1097/01.asn.0000033611.79556.ae

[25] Lan HY (2011) Diverse roles of TGF-beta/Smads in renal fibrosis and inflammation. International journal of biological sciences 7: 1056–1067. 21927575 10.7150/ijbs.7.1056 PMC3174390

[26] Kielar ML, John R, Bennett M, Richardson JA, Shelton JM, et al (2005) Maladaptive role of IL-6 in ischemic acute renal failure. J Am Soc Nephrol 16: 3315–3325. 16192425 10.1681/ASN.2003090757

[27] Nechemia-Arbely Y, Barkan D, Pizov G, Shriki A, Rose-John S, et al (2008) IL-6/IL-6R axis plays a critical role in acute kidney injury. J Am Soc Nephrol 19: 1106–1115. 18337485 10.1681/ASN.2007070744 PMC2396933

[28] Powell DW, Mifflin RC, Valentich JD, Crowe SE, Saada JI, et al (1999) Myofibroblasts. I. Paracrine cells important in health and disease. Am J Physiol 277: C1–9. 10409103 10.1152/ajpcell.1999.277.1.C1

[29] Eddy AA (2005) Progression in chronic kidney disease. Advances in chronic kidney disease 12: 353–365. 16198274 10.1053/j.ackd.2005.07.011

[30] Zhang G, Moorhead PJ, el Nahas AM (1995) Myofibroblasts and the progression of experimental glomerulonephritis. Experimental nephrology 3: 308–318. 7583053

[31] Roberts IS, Burrows C, Shanks JH, Venning M, McWilliam LJ (1997) Interstitial myofibroblasts: predictors of progression in membranous nephropathy. Journal of clinical pathology 50: 123–127. 9155692 10.1136/jcp.50.2.123 PMC499736

[32] Essawy M, Soylemezoglu O, Muchaneta-Kubara EC, Shortland J, Brown CB, et al (1997) Myofibroblasts and the progression of diabetic nephropathy. Nephrology, dialysis, transplantation: official publication of the European Dialysis and Transplant Association – European Renal Association 12: 43–50. 10.1093/ndt/12.1.43 9027772

[33] Matloubian M, David A, Engel S, Ryan JE, Cyster JG (2000) A transmembrane CXC chemokine is a ligand for HIV-coreceptor Bonzo. Nat Immunol 1: 298–304. 11017100 10.1038/79738

[34] Garcia GE, Truong LD, Li P, Zhang P, Johnson RJ, et al (2007) Inhibition of CXCL16 attenuates inflammatory and progressive phases of anti-glomerular basement membrane antibody-associated glomerulonephritis. Am J Pathol 170: 1485–1496. 17456756 10.2353/ajpath.2007.060065 PMC1854945

[35] Zhang L, Ran L, Garcia GE, Wang XH, Han S, et al (2009) Chemokine CXCL16 regulates neutrophil and macrophage infiltration into injured muscle, promoting muscle regeneration. Am J Pathol 175: 2518–2527. 19893053 10.2353/ajpath.2009.090275 PMC2789607

[36] Cheever AW, Williams ME, Wynn TA, Finkelman FD, Seder RA, et al (1994) Anti-IL-4 treatment of Schistosoma mansoni-infected mice inhibits development of T cells and non-B, non-T cells expressing Th2 cytokines while decreasing egg-induced hepatic fibrosis. Journal of immunology 153: 753–759. 8021510

[37] Finkelman FD, Wynn TA, Donaldson DD, Urban JF (1999) The role of IL-13 in helminth-induced inflammation and protective immunity against nematode infections. Current opinion in immunology 11: 420–426. 10448138 10.1016/S0952-7915(99)80070-3

[38] Wynn TA (2008) Cellular and molecular mechanisms of fibrosis. J Pathol 214: 199–210. 18161745 10.1002/path.2277 PMC2693329

